# The Intercolonial Medical Congress of Australasia

**Published:** 1888-02

**Authors:** 


### THE INTERCOLONIAL MEDICAL CONGRESS OF AUSTRALASIA.

The first Intercolonial Medical Congress was opened in Adelaide on August 30, under the Presidency of J. C. Verco, M. D. (Lond.), F. R. C. S., Eng.

*Secretary's Report.*—Dr. Poulton read the report, which stated that this Intercolonial Medical Congress of Australasia was the outcome of a suggestion made by the council of the South Australian Branch of the British Medical Association at the annual meeting held in June, 1886, that a medical congress might well take place during the jubilee of Her Majesty's reign, at a time when the colony of South Australia would be celebrating the completion of the first fifty years of its history, and during the term of the International Exhibition in Adelaide. The members of the branch heartly concurred in the suggestion, and being the only organized medical society in South Australia, forthwith appointed a committee to co-operate with the council in formulating the scheme of such a congress, and in inviting the co-operation of the profession throughout the Australasian colonies. The joint provisional committee consisted of Messrs. Cleland, Clindening, Corbin, Hayward, and Drs. Gardner, Poulton, Stirling, Davies Thomas, Verco, and Watson. Numerous promises of support were received from representative members of the profession in the various colonies, and the council of the University of Adelaide generously granted the use of their halls. The presidents of all the medical societies of Australasia had become vice-presidents of the congress. Special delegates were present from the New Zealand Medical Association, from the Universities of Sydney and Melbourne, and from the Medical Societies of Sydney, Melbourne, Brisbane, Ballarat, and Sandhurst. The number of members present was 155, and included practitioners from New South Wales, Victoria, New Zealand, Queensland, South Australia, the Northern Territory, and Fiji. The work of the congress would be conducted in the four main sections — medicine, surgery, gynæcology, and state medicine — under the presidency of Dr. Williams, Mr. Fitzgerald, Mr. Foreman, and Dr. Whittell, respectively.

*Official Welcome.*—His Excellency, the governor, then welcomed the Congress to Adelaide in an appropriate speech.

*President's Address.*—The President (Dr. Verco) delivered an address, in which he mentioned the various reasons why Australian professional men should meet in congress; these were especially that they might make common property of the facts peculiar to Australia which had been observed in disease, of the methods which had proven most appropriate in their hands, of the new remedies nature had provided, of suggestions relating to medical ethics or legal status arising out of colonial life, and finally that an opportunity might be afforded to those far away to contribute their unique or exceptional experiences for the general interest, and to reap the pleasure and profit of that social and professional intercourse which was so rarely theirs. He dwelt on the pharmaceutical and balneological novelties which Australasia might be able to afford, and on the climatological peculiarities which might not only be of use in the treatment of disease, but were actually modifying the physical type. "I have," he said "measured 300 South Australasian immigrants from the old world; they stand 5 ft. 7.13 in., and weigh 146.58 lbs. I have contrasted with them 259 South Australians born in this colony; they average 5 ft. 8.21 in., and weigh 146.42 lbs. Our native population are therefore exactly an inch taller than their forefathers, and within the fraction of a pound the same weight. What is the significance of these facts? That our southern climate, our social circumstances, our mode of life, are altering the physical constitution of the healthy man, of the growing child, and giving him a taller and more slender form."

*Votes of Thanks.*—A vote of thanks to the governor was proposed by Dr. Stirling and seconded by Dr. Wilkinson, M. L. A. (Sydney), and duly acknowledged. A vote of thanks to the President for his able address was moved by Archdeacon Farr, and seconded by the chief justice.

SECTION OF MEDICINE.

*President's Address.*—Owing to the illness of Dr. Williams, his address on the advances of modern medicine was read by Professor Allen. The chief feature of modern medical work was the attempt to grasp the natural history of disease. Dr. Williams thought that a thorough sympathy, an active co-operation in inquiry, between physician and pathlogist, which shall lead each to assist, to verify, to correct the work of the other, were needed. The causes of disease, the influence of family tendency and of personal surroundings, the connected history of patients from birth to death, the questions of idiosyncracy in treatment, all these formed the domain of inquiry for the private practitioner. In these matters, too, the county physician had more accurate and continuous knowledge of his patient; he dealt with questions in simpler form, and hence his observations might be most valuable. Without doubt, the present isolated professional life of the bulk of country practitioners involved a grievous waste of useful knowledge which should be conserved for the general good. He expressed approval of the system of collective investigation.

*Papers.*—During the Congress the following papers were read in this section: Fear as a Factor in Producing many of the Alarming Symptoms following the Bite of Australian Snakes, by the Hon. J. M. Creed, M. L. C. (Sydney); Some Remarks upon the South Australian Climates, and their Influence on Phthisis, by Dr. Astles, M. D. (Adelaide); on Grindelia Robusta, by Dr. Dixon, M. B., Lecturer on Materia Medica at the Sydney University; Hydatid Disease of Brain, J. Davies Thomas, M. D.; Tuberculosis, F. W. Elsner, F. R. C. S. I.; Hydatid Disease (a case), J. W. Springthorpe, M. D.; Anæmia, J. Reid, M. D.; Epilepsy (cases), J. W. Springthorpe, M. D.; Dilatation of Stomach, F. W. Elsner, F. R. C. S. I. Some interesting notes on modification of symptoms in Central Australia were laid before the Congress by Mr. J. P. Baker, F. R. C. S., of Strangways, South Australia.

*New Zealand Thermal Waters.*—A paper on the Thermal Springs District of New Zealand and the Government Sanitarium at Rotorua was submitted by Dr. Ginders, of New Zealand. After dilating on the delightful climate of Rotorua, Dr. Ginders mentioned that the thermal springs district of New Zealand comprised an area close on 1,000 square miles, and its altitude ranged from 1,000 to 2,000 feet above sea-level. No spring had obtained a higher reputation than that known as the Priest's Bath. The character of the water was sulphurous, aluminous, and strongly acid; and its temperature varied from 98° to 106°.

### SECTION OF SURGERY.

*President's Address.*—The address to the Surgical Section was delivered by Mr. T. N. Fitzgerald. After referring to the extraordinary advances made in medicine within the memory of living men, he said Australian surgery possessed two peculiar features—(1), the prevalence of hydatid disease; and (2), the absence of rachitis. A student might go through the whole of his curriculum without witnessing a genuine case of rickets. Professor MacEwen, in Glasgow, a town whose population was not greater than that of Victoria, recorded his thousandth operation for genu valgum. Mr. Fitzgerald ventured to say that, if the whole colony was searched, not five cases suitable for operation would be found. The Australian child, even emerging from the lowest den, was straight-limbed and level-featured. A neglected club-foot might possibly be met with, and occasionally chronic hip-disease was seen; but rachitis and bone distortions arising from other causes than fracture were almost unknown.

*Papers.*—The following papers were among those read in this Section: Case of Scrofulous Pyelonephritis, Removal of Kidney by Langenbeck's Incision; Lumbar Drainage, Recovery, by Dr. H. W. Mansell, M. D. T.C.D., Honorary Surgeon of the Dunedin Hospital; Hydronephrosis, Removal of Kidney, Recovery, by Dr. Jell, Wellington, New Zealand (read by Dr. Closs). Demonstration and illustration, by Dr. Mansell, on (*a*) the use of carver's tools in hard cleft palate; (*b*) a demonstration enterectomy; (*c*) extroversion of bladder; (*d*) removal of foreign body from the bladder by suprapubic operation; (*e*) a method of radical cure of femoral hernia. Extirpation of Larynx, by Dr. W. Gardner; A New Method of Drainage after Abdominal Incision, by Mr. O'Hara; Ophthalmic Papers (two), by Dr. Symons; On Operation in Compound Depressed Fracture of the Skull, by Mr. Muskett.

### SECTION OF GYNÆCOLOGY.

*President's Address.*—The address was delivered by Dr. Forman, of New South Wales.

*Papers.*—The following papers were read: History and Progress of Ovariotomy in the Australian Colonies, by Dr. Pinnock, M.B., etc, (Ballarat); Oöphoritis and its Relation to Oöphorectomy, by Dr. J. O. Closs; On Shortening the Round Ligaments, by J. Foreman, M.R.C.S.; The Alexander-Adams Operation, by Dr. W. Gardner.

### SECTION OF STATE MEDICINE.

*President's Address.*—Dr. Whittell referred first of all to the evidence taken by the commission appointed by the New South Wales Government to inquire into the law respecting the practice of medicine and surgery in that colony, and the revelations and confessions were astounding. He then discussed the means of preventing the importation of infectious diseases. The Northern Territory and Queensland, he observed, lay dangerously near to thickly populated countries which were seldom or never free from small-pox. The Chinese from those countries kept up a constant immigration into the northern ports, and it was possible that a patient might become infected with small-pox in Hong-Kong or Sumatra, might sail to the Northern Territory, land there, mix with the population, and be lost sight of before the outward signs of the disease became manifest. He said that a station was required at Port Darwin, where the strictest investigation should be made, and where no vessel should be allowed to pass on until all risk of its bringing infection should have ceased. He also pointed out that it would not be safe to trust to inspection alone, but that vaccination ought to be systematically performed.

*The State, the Practitioner, and the Public.*—Dr. Stirling delivered an address, in which he stated that though to some extent the contentions of the profession through the medical associations in Great Britain and Australia to induce the legislature to undertake the control over certain matters in which the medical profession were peculiarly interested, had been carried into effect, there were still many points on which practitioners had not received the consideration they deserved. He urged, first, that the state should accord protection to qualified practitioners only, and should at least discourage, if it did not entirely prohibit, practice by unqualified persons; secondly, that the assumption of professional titles and qualifications by persons possessing none should be punishable; thirdly, that medical certificates as to the cause of death should be received only from duly qualified medical men; fourthly, the definition of a qualified practitioner should be more strict; fifthly, that the authority which determined the issuing of a certificate of registration as a legally qualified practitioner, should equally have the power of canceling or suspending them.

Dr. Bickle, in a paper on the Relation of the Profession to the Public, referred especially to the abuse of clubs.

Dr. Abramowski agreed with Dr. Stirling's suggestions.

Dr. Jameson said that a person desiring to practice in Victoria was obliged to produce satisfactory credentials of having studied a proper length of time, and that he possessed the necessary qualifications, before he was registered.

The Hon. Dr. Creed said that in New South Wales practically no law to control the practice of medicine existed. Quacks could not be prevented from practicing, but people should be informed exactly with whom they were dealing.

*Tests of Vision.*—Dr. James Rudall delivered an address on government responsibility in regard to sight-testing for land and sea service, and gave as his conclusions that it was the duty of the government to pass laws which could not be evaded, to ensure the public safety in traveling.

*Cremation.*—The Hon. Dr. Creed, after referring to the advantages of cremation, said that he thought the object of the advocates of cremation would be best carried out by the establishment of societies for the purpose in all the colonies. The duty of such societies would be to detect and bring before the public any instances of danger to health which had been created by the burial of bodies in unsuitable cemeteries; to disseminate literature giving information on the subject amongst the people; to provide, in due time, fitting apparatus for carying out the process; to advocate the passing of laws to ensure its being done without shock to the sensibilities of those persons whose prejudices prevented their being favorable to it, and to provide regulations which would prevent it, in some rare instances, being made a means for the concealment of crime. In this way public thought would be so educated by example that in a very few years cremation would become as general and as much desired by the majority as burial is at present. A danger to health would be removed, and he thought they would agree with him in believing that refined sentiment would be better conserved than it was at present.

*Entertainments.*—In the afternoon of the first day (August 30) the mayor of Adelaide entertained the members of the Congress at luncheon, and in the evening a *conversazione* was held in the University. On September 2 (the last day of the Congress), the President and South Australian members entertained the visiting members and many distinguished guests at a public dinner.

*Next Congress.*—It was decided that the next Congress be held in Melbourne, in 1890, or at such time as the medical societies of Victoria shall determine. The Hon. Dr. Creed (Sydney) proposed that Mr. T. N. Fitzgerald, of Melbourne, should be the President-elect of the Congress, and in doing so paid a tribute to the value of Mr. Fitzgerald's work. Dr. Gardner (Adelaide), in seconding, said he knew of no gentleman who was more fitted to hold the position. The motion was carried with acclamation. Mr. T. N. Fitzgerald (the President-elect) briefly thanked the meeting for appointing him to such a responsible position, which he would undertake with great pleasure.

